# The perceived effects of COVID-19 pandemic on female genital mutilation/cutting and child or forced marriages in Kenya, Uganda, Ethiopia and Senegal

**DOI:** 10.1186/s12889-022-13043-w

**Published:** 2022-03-29

**Authors:** Tammary Esho, Dennis J. Matanda, Timothy Abuya, Sintayehu Abebe, Yeshitila Hailu, Khaltoume Camara, Bachir Mouhammed, Tonny Kapsandui, Lilian Kamanzi, Andrew Wabwire, Patrick Kagurusi, Maureen Nankanja, Anne Gitimu, David Kawai, John Kogada, Millicent Ondigo, Joachim Osur

**Affiliations:** 1 Amref Health Africa, Langata Rd, PO Box 27691-00506, Nairobi, Kenya; 2 Population Council, Avenue 5, 3rd Floor Rose Ave, Nairobi, Kenya; 3 Amref Health Africa, Addis Ababa, Ethiopia; 4 Amref Health Africa, Dakar, Senegal; 5 Amref Health Africa, Kampala, Uganda; 6 Amref International University, Nairobi, Kenya

**Keywords:** Perceived effects, COVID-19, Female genital mutilation/cutting, Child or forced marriage

## Abstract

**Background:**

The effects of COVID-19 on harmful traditional practices such Female Genital Mutilation/Cutting (FGM/C) and Child or Forced Marriages (CFM) have not been well documented. We examined respondents’ perceptions on how the COVID-19 pandemic has affected FGM/C and CFM in Kenya, Uganda, Senegal, and Ethiopia.

**Methods:**

A cross-sectional study design with a mixed methods approach was used. Data collection on participants’ perceptions on the effects of COVID-19 on FGM/C and CFM took place between October-December 2020. Household surveys targeting women and men aged 15–49 years in Kenya (*n = 312*), Uganda (*n = 278*), Ethiopia (*n = 251*), and Senegal (*n = 208*) were conducted. Thirty-eight key informant interviews with programme implementers and policymakers were carried out in Kenya (*n = 17*), Uganda (*n = 9*), Ethiopia (*n = 8*), and Senegal (*n = 4*).

**Results:**

In Kenya, the COVID-19 pandemic has contributed to the increase in both FGM/C and CFM cases. Minimal increase of FGM/C cases was reported in Uganda and a significant increase in CFM cases. In Ethiopia, the COVID-19 pandemic had a limited perceived effect on changes in FGM/C and CFM. In Senegal, there were minimal perceived effects of COVID-19 on the number of FGM/C and CFM cases. The pandemic negatively affected implementation of interventions by the justice and legal system, the health system, and civil societies.

**Conclusions:**

The pandemic has had varied perceived effects on FGM/C and CFM across the four countries. Generally, the pandemic has negatively affected implementation of interventions by the various sectors that are responsible for preventing and responding to FGM/C and CFM. This calls for innovative approaches in intervening in the various communities to ensure that women and girls at risk of FGM/C and CFM or in need of services are reached during the pandemic. Evidence on how effective alternative approaches such as the use of call centres, radio talk shows and the use of local champions as part of risk communication in preventing and responding to FGM/C and CFM amid COVID-19 is urgently required.

**Supplementary information:**

The online version contains supplementary material available at 10.1186/s12889-022-13043-w.

## Introduction

Female Genital Mutilation/Cutting (FGM/C) and Child or Forced Marriage (CFM) violates human rights with far-reaching negative effects on the health of women [1–3]. FGM/C involves partial or total removal of the external female genitalia or injury to the female genital organs for non-medical reasons [4]. Nearly 200 million women and girls in 30 countries have undergone FGM/C [5, 6], while approximately 70 million girls aged 0–14 years have been cut or at risk of being cut [7]. Although there are variations in the definitions of age at maturity, CFM is defined as marriage before the age of 18 years [8] or when marriage is forced or arranged [9, 10]. In 30 countries of sub-Saharan Africa, approximately 30% of girls have experienced CFM [11]. Annual estimates indicate that about 15 million girls are married off before their 18th birthday, and if strategic interventions are not implemented, this would increase to 1.2 billion by 2050 [11]. FGM/C and CFM are influenced by community and family interests on sustaining cultural or religious beliefs such as purity, honour, fidelity in marriage, preserving virginity before marriage and financial security [12]. The two practices may coexist or occur independently [13, 14].

Due to the complex relationship of the two practices, global public health challenges like the Coronavirus Disease 2019 (COVID-19) pandemic are likely to exacerbate such practices. A handful of studies have examined the impact of COVID-19 on gender-based violence [15, 17, 18], while fewer studies have examined its impact on harmful traditional practices such as FGM/C and CFM [18]. The goal of this study was therefore to bring to the fore the perceived effects of COVID-19 pandemic on harmful traditional practices in sub-Saharan Africa. Specifically, we conducted a multi-country study to generate evidence on the perceived effects of COVID-19 pandemic on FGM/C and CFM in Kenya, Uganda, Ethiopia, and Senegal.

## Methods

This was a cross-sectional mixed methods study that employed quantitative and qualitative approaches in data collection and analysis. The quantitative aspect involved conducting a household survey targeting women and men aged 15–49 years. The qualitative method used in-depth interviews (IDI) to collect data from programme implementers and policymakers with a history of working on FGM/C and CFM in the four focus countries. The national estimates of FGM/C and CFM in the four countries are: Kenya (21% and 23%), Uganda (0.3% and 34%), Ethiopia (65% and 40%) and Senegal (24% and 31%) respectively [19–22]. Since the sub-national estimates of FGM/C and CFM varies considerably within the countries, data was collected in areas where both practices are prevalent. In Kenya, data was collected in Kajiado, Samburu and Marsabit counties; Bukwo district in Uganda; Qewot district in Ethiopia, and Sedhiou municipality in Senegal.

All research assistants with a professional background in social sciences underwent a two-day online training. During the training, research assistants were introduced to the study including the objectives of the study, data collection processes, and ethical considerations during and after the research. The training included focused sessions and exercises regarding the meaning and process of informed consent as well as the importance of protecting the privacy of subjects, and confidentiality of the information obtained. Data collection tools were pre-tested to ensure consistency and identify potentially sensitive questions that were modified accordingly. Ethical approval was obtained from relevant country specific ethical review boards: Kenya (Amref Ethics and Scientific Review Committee – ESRC P826-2020), Uganda (Makerere University, College of Health Sciences – Protocol 881), Ethiopia (Ethiopian Public Health Association – EPHA/OG/501/20) and Senegal (Comité nationale d’éthique pour la recherche en santé – 00 000 232). All participants consented either in writing or verbally prior to participating in the study. Data collection took place in the months of October to December 2020.

### Household survey

The quantitative survey involved collecting data from women and men aged 15–49 years from ethnic groups that have traditionally practised FGM/C and CFM and resided in the study sites for at least five years. This selection criterion was important in ensuring that study respondents had a more reliable assessment of the changes in FGM/C and CFM before and during the pandemic. The specific ethnic groups sampled included: the Maasai, Samburu and Borana in Kenya; the Sabiny/Sebei in Uganda; Poular, Mandingue, Balante and Diola in Senegal; and Oromo and Amhara in Ethiopia. Once the sub-regions were selected, we listed the lowest administrative areas and randomly selected villages where data collection took place. Since the effect of COVID-19 on FGM/C and CFM is not known, a 50% prevalence with a 10% precision was used for sample size calculation. A total of 1,049 respondents were interviewed: Kenya (*n* = 312), Uganda (*n* = 278), Ethiopia (*n* = 251), and Senegal (*n* = 208). A team of research assistants reached out to potential participants while observing COVID-19 safety guidelines and sought their consent or assent to be interviewed. We collected data on participants’ perceptions on the trends of FGM/C and CFM cases before and during the COVID-19 pandemic; and perceptions on the role of judicial system, healthcare workers and the civil society in responding to FGM/C and CFM during the pandemic.

### Key informant interviews

The qualitative component included key informant interviews (KIIs) with programme implementers and policymakers who have been involved in supporting interventions to end FGM/C and CFM. Convenience sampling was used to identify representatives of international and local organisations and government representatives who provide support towards ending FGM/C and CFM as well as those supporting interventions to contain the spread of COVID-19. A total of 17 respondents were interviewed in Kenya (9 policymakers and 8 programme implementers); 9 respondents in Uganda (6 policymakers and 3 programme implementers); 8 respondents in Ethiopia (3 policymakers and 5 programme implementers); and 4 respondents in Senegal (2 policymakers and 2 programme implementers). Participants were interviewed in languages they were fluent in by research assistants with training in qualitative data collection using a guide. The interviews were audio-recorded with consent of the participant.

### Data processing and analysis

Research assistants were trained to encode notes by encrypting devices, to anonymise the participants and categorize the subjects according to their interview types. Notes were translated and linked to a database management program (NVivo 12). Audio recordings of KIIs were translated and transcribed verbatim. In reviewing text data from interviews, inductive analysis was used to identify themes and patterns and construct typologies. The inductive analysis involved the principal investigator and members of the data analysis team independently coding the first two transcripts, which were compared, and differences reconciled. Codes corresponding to themes and constructs were used to organize data for refined analysis. The research team met regularly to discuss, add new codes, or sub-codes, and to identify emerging themes. Analysis of quantitative data entailed descriptive statistics. Chi-square tests and significance tests of proportions were conducted to determine if differences between sub-groups were statistically significant with probability set at 0.05. Please see Additional file 1: Appendix 1, 2 and 3 for details on the questions asked during the interviews.

## Results

### Background characteristics of respondents

Table 1 summarizes key sociodemographic characteristics of respondents who participated in the household survey in the four countries. There were more women sampled than men (this was deliberate as FGM/C and CFM directly affects women than men) with majority of respondents residing in rural areas. In Kenya and Ethiopia, majority of respondents were aged between 26 and 35 years, while in Uganda and Senegal, the majority were aged between 15 and 18 years. Apart from Uganda where most of the respondents had incomplete secondary education, most of the respondents in the other three countries had no education. Across all the countries, most of the respondents were married.

**Table 1:** Sociodemographic characteristics of respondents in the community survey

Characteristics	Kenya		Uganda		Ethiopia		Senegal	
	**N**	**%**	**N**	**%**	**N**	**%**	**N**	**%**
**Gender**								
Male	65	20.8	52	18.7	49	19.5	59	28.4
Female	247	79.2	226	81.3	202	80.5	149	71.6
**Location of respondent**								
Urban area	12	3.8	0	0.0	7	2.8	93	44.7
Rural setting	300	96.2	278	100.0	244	97.2	115	55.3
**Age of respondent (years)**								
15–18	70	22.4	143	51.4	56	37.6	55	36.9
19–25	54	17.3	35	12.6	68	45.6	46	30.9
26–35	108	34.6	32	11.5	78	52.3	49	32.9
36–45	54	17.3	46	16.5	41	27.5	46	30.9
Above 45	26	8.3	22	7.9	8	5.4	12	8.1
**Level of education**								
No education	162	51.9	74	26.6	173	68.9	68	32.7
Completed primary education	43	13.8	84	30.2	29	11.6	20	9.6
Incomplete secondary education	92	29.5	108	38.8	44	17.5	63	30.3
Completed secondary and/or higher education	15	4.8	12	4.3	5	2.0	57	27.4
**Marital status**								
Married	196	62.8	175	62.9	178	70.9	120	57.7
Separated	8	2.6	4	1.4	0	0.0	0	0.0
Divorced	6	1.9	1	0.4	8	3.2	1	0.5
Widowed	13	4.2	0	0.0	11	4.4	2	1.0
Single	89	28.5	98	35.3	54	21.5	85	40.9
**Total**	**312**	**100.0**	**278**	**100.0**	**251**	**100.0**	**208**	**100.0**

### FGM/C and CFM cases before and during COVID-19

*Findings from quantitative interviews* with community members on FGM/C and CFM cases before and during COVID-19 are shown in Table 2. In Kenya, before COVID-19, majority of the respondents were of the view that cases of FGM/C and CFM were decreasing in Kajiado, Samburu and Marsabit counties. In contrast, during COVID-19, most of the study respondents believed that the pandemic had led to an increase in both FGM/C and CFM cases. The most common reason given for the increasing number of FGM/C cases was closure of schools (50%), people staying at home for longer including potential victims (25%) and economic losses (39%).

**Table 2:** Perceived status of FGM/C and CEFM cases before and during COVID-19

	Kenya			Uganda			Ethiopia			Senegal		
	**N**	**%**	***P*-value**	**N**	**%**	***P*-value**	**N**	**%**	***P*-value**	**N**	**%**	***P*-value**
**Percentage of respondents reporting that FGM/C cases before COVID-19 were**:												
Same as now	49	15.7	< 0.001	1	0.4	0.216	150	59.8	0.837	13	6.3	0.22
Decreasing	196	62.8		257	92.4		69	27.5		138	66.3	
Increasing	48	15.4		14	5.0		32	12.7		22	10.6	
Don’t know/ No response	19	6.1		6	2.2		0	0.0		35	16.8	
**Percentage of respondents reporting that FGM/ cases during COVID-19 were**:												
Same as now	51	16.3	< 0.001	30	10.8	0.062	204	81.3	0.126	22	10.6	0.592
Decreasing	81	26.0		228	82.0		46	18.3		134	64.4	
Increasing	171	54.8		19	6.8		1	0.4		5	2.4	
Don’t know/ No response	9	2.9		1	0.4		0	0.0		47	22.6	
**Percentage of respondents reporting that CEFM cases before COVID-19 were**:												
Same as now	42	13.5	< 0.001	3	1.1	< 0.001	124	49.4	0.655	17	8.2	0.423
Decreasing	193	61.9		234	84.2		75	29.9		129	62.0	
Increasing	67	21.5		40	14.4		52	20.7		27	13.0	
Don’t know/ No response	10	3.2		1	0.4		0	0.0		35	16.8	
**Percentage of respondents reporting that CEFM cases during COVID-19 were**:												
Same as now	45	14.4	< 0.001	10	3.6	0.111	175	69.7	0.712	26	12.5	0.732
Decreasing	66	21.2		74	26.6		60	23.9		124	59.6	
Increasing	198	63.5		193	69.4		16	6.4		27	13.0	
Don’t know/ No response	3	1.0		1	0.4		0	0.0		31	14.9	
**Total**	**312**	**100.0**		**278**	**100.0**		**251**	**100.0**		**208**	**100.0**	

In Uganda, before the pandemic, most of the respondents reported that cases of FGM/C and CFM were decreasing. During COVID-19, there was a slight increase in the proportion of community members who believed that the pandemic had led to a slight increase in FGM/C cases (from 5 to 7%) and a substantial increase in CFM cases (from 14 to 69%). The common reason given for the increasing number of FGM/C cases was people staying at home for longer including potential victims (50%); while loss of income (59%) was the most common reason given for perceived increase in CFM cases during COVID-19.

In Ethiopia, majority of the respondents were of the view that there were no changes in cases of FGM/C and CFM before COVID-19. The situation was the same during COVID-19 with most of the study respondents believing that the number of FGM/C and CFM cases had not changed. For the few who believed that the number of CFM cases were increasing during the pandemic, the most common reason given was people staying at home for longer including potential victims (80%).

In Senegal, before COVID-19, most of the respondents were of the view that cases of FGM/C and CFM were decreasing. During the pandemic, a bigger proportion of survey respondents were of the view that the number of FGM/C cases were increasing with the most common reason given being people staying at home for longer including potential victims (60%). With regards to CFM, the most common reason given for the increasing cases was reduced efforts in programmes supporting potential victims (26%).

*Findings from qualitative interviews* with policymakers and programme implementers in Kenya showed convergence of opinion with community members regarding the perceived increase in FGM/C and CFM cases during the pandemic. The key informants noted that the perceived increase was likely to be associated with lack of protection/safe spaces for girls that is often provided by schools and rescue centres. In addition, they noted that the perceived increase was likely to be perpetuated by stigma that is associated with teenage pregnancy leading to CFM. The following quotes are worth noting:

*“Lack of enforcement and protection, especially those from the boarding schools now, as well as the rescue centres. Since girls are at home, the protection is no more. They are out there, there is no monitoring…they have ample time to practise FGM and child marriage.”* Programme Implementer #1, Kenya.

*“From the cases that I know, you hear a girl has been married off and then you hear that she was already pregnant. So, it is like the marriage was to avoid the stigma. In our community when a girl gives birth and she is not married then you have that kind of stigma that you are a bad girl, you do not have discipline. So, I think to avoid the stigma that comes with having a baby without a husband, then the moment a girl is pregnant they are married off.”* Policymaker #3, Kenya.

In Uganda, key informants were ambivalent on the status of FGM/C in the study area. Some respondents were of the view that cases had increased and were being performed in secret while others believed that the cases had not increased as the season for cutting (December holidays—festive month when schools have closed) had not yet reached. The following quotes exemplify these views:

*“It is increasing, the early marriage compared to the FGM as the law is in place and a few people tend to escape and cut themselves in the bushes. As much as they have tried always to bring in the interventions, but they are still doing it [FGM/C] but not openly. The early marriages are common because the girl child is not going to school.”* Programme Implementer #1, Uganda.

*“Beware that this is the circumcision year and we have not come to the end of the year more so to the climax because it is mostly in December… We do not know what is going to happen between now and December because since the year begun, I have not come across a girl who has been cut, or any woman who has been mutilated.”* Policymaker #2, Uganda.

The protective effect of schools and the complex interaction of poverty, FGM/C and CFM was evident from discussions as noted in the following excerpts:

*“Initially when children were going to school, they had no time but being at home they are idle, and I believe that is one of the key reasons. I believe when children are at school these cases of early pregnancies can be minimized.”* Programme Implementer #4, Uganda.

*“Because of COVID-19, there was a lot of poverty because people were locked down. So, when someone gets into such a problem, they would want to go and negotiate [for bride price] because they know at the end of it, they will get something [money]. Which also forces some of them to marry off their daughters early.”* Policymaker #1, Uganda.

In Ethiopia, findings from the community survey appeared to contradict results from KIIs with policymakers and programme implementers who believed that there was perceived increase in cases of CFM due to closure of schools but were uncertain about changes in FGM/C cases. A policymaker reported that the steering committee responsible for children and gender issues had reviewed local data on reported cases of CFM and FGM/C and noted an increase in CFM cases but not in FGM/C cases.

*“Reports on CFM are coming from police officers, health extension workers, health development army but FGM/C cases are not that much as compared to CFM. We have reviewed the report with the steering committee from police office, health, judiciary, education, and other offices including NGO’s [Non-Governmental Organizations] and FBO [Faith Based Organizations]…we received more cases of child marriage.”* Policymaker #1, Ethiopia.

Nonetheless, other key informants noted that FGM/C was practised discreetly, and stakeholders had shifted their focus on COVID-19 more than FGM/C and CFM and therefore it may be difficult to report such cases. COVID-19 containment measures including restriction of movement could also have affected reporting from the community and key government agencies as explained in the following excerpt:

*“After Covid cases were identified in our country, it was very difficult to support and sensitize communities by going house to house. People are focusing on COVID-19 than FGM and CFM. As a result, we might have missed information about CFM and FGM. Since communities’ movement was restricted by Covid, police officers might not receive report from health extension workers, and health development army on what is happening in communities.”* Programme Implementer #2, Ethiopia.

In Senegal, programme implementers and policymakers observed that there was perceived increase in the number of FGM/C and CFM cases due to the COVID-19 restrictions. They were of the view that due to the implementation of COVID-19 prevention guidelines; perpetrators of FGM/C were conducting the practice in secret. Due to closure of schools, girls were more at risk of getting pregnant which would lead to CFM.

*“COVID-19 has blocked all ongoing activities and slowed progress in achieving results. This in return has led to a resurgence of the practice [FGM/C] … Cutters can excise without people knowing because the practice is done at a very young age; even before the first birthday. This is not controlled because of the COVID-19 restrictions.”* Programme Implementer #1, Senegal.

There was also the perception that resources were focussed on COVID-19 at the expense of CFM and FGM/C, and therefore a resurgence in these harmful practices. Other reasons mentioned included lack of monitoring mechanisms that would allow tracking and reporting of FGM/C and CFM cases.

### Adequacy of the justice and legal system in addressing FGM/C and CFM

*Quantitative survey respondents* were asked to rate the response of the justice and legal system in addressing FGM/C and CFM during the COVID-19 pandemic (Table 3). In Kenya, findings showed that over 60% of community members considered the justice and legal system’s response to FGM/C and CFM cases to be either poor or average. The main barrier to the justice and legal system to respond effectively during the pandemic was inadequate reporting by victims (46%) or challenges of accessing victims due to restrictions and fear of lack of services being offered.

**Table 3:** Legal system’s response in addressing FGM/C and CEFM during COVID-19

	Kenya			Uganda			Ethiopia			Senegal		
	**N**	**%**	***P*-value**	**N**	**%**	***P*-value**	**N**	**%**	***P*-value**	**N**	**%**	***P*-value**
**Response of the legal system on FGM/C during COVID-19**												
Poor	87	27.9	< 0.001	63	22.7	0.003	9	3.6	0.684	61	36.7	0.794
Average	124	39.7		72	25.9		4	1.6		56	33.7	
Good	86	27.6		143	51.4		236	94.8		49	29.5	
Excellent	13	4.2		0	0.0		0	0.0		0	0.0	
Don’t know	2	0.6		0	0.0		0	0.0		0	0.0	
**Response of the legal system on CEFM during COVID-19**												
Poor	86	27.6	< 0.001	66	23.7	0.133	9	3.7	0.089	63	33.9	0.942
Average	126	40.4		64	23		14	5.7		57	30.6	
Good	82	26.3		148	53.2		223	90.7		66	35.5	
Excellent	16	5.1		0	0.0		0	0.0		0	0.0	
Don’t know	2	0.6		0	0.0		0	0.0		0	0.0	
**Total**	**312**	**100.0**		**278**	**100.0**		**251**	**100.0**		**208**	**100.0**	

In Uganda, community members’ opinions were divided with slightly over half of the respondents being of the view that the justice and legal system’s response to FGM/C and CFM was good, while slightly less than half believing that its response to FGM/C and CFM was either poor or average. The main barrier to the justice and legal system to respond effectively during the pandemic was inadequate reporting by victims (40%) and challenges of accessing victims due to restrictions (28%) and fear of lack of services being offered (10%).

In Ethiopia, over 90% of community members considered the justice and legal system’s response to FGM/C and CFM to be good while less than 10%, believed that the justice and legal system’s response to FGM/C and CFM was either average or poor. The main barrier to the justice and legal system to respond effectively during the pandemic was inadequate reporting by victims (41%), fear of lack of services being offered (21%) and challenges of accessing victims due to restrictions (8%).

In Senegal, over 60% of community members considered the justice and legal system’s response to FGM/C and CFM cases to be either poor or average. The main barrier to the justice and legal system to respond effectively during the pandemic was inadequate reporting by victims (40%), fear of lack of services being offered (26%) and challenges of accessing victims due to restrictions (16%).

*Qualitative data from key informant interviews* showed that in Uganda, challenges facing the legal and judicial system during COVID-19 included restrictions in movement which made it impossible to conduct court sessions, lack of reporting from community members and corruption within the police.

*“When you look at the judicial system, when COVID-19 came or when we went into this period [lockdown], some activities like court hearing were limited, and in some places, there were no staff, we did not have public transport… Now it means that people who were to attend court, the people who were to get justice from court could not access it.”* Policymaker #3, Uganda.

*“When you look at the police…they have been moving around but, you know police they deal with issues that have been reported, so under COVID-19, you find that many issues were not reported because community members are afraid. The police are also compromised at the community level… the police do not have the guts to reach out to them unless they get the reports.”* Policymaker #2, Uganda.

In Ethiopia, policymakers and programme implementers detailed how the justice and legal system had rolled out strategies to deal with FGM/C and CFM during COVID-19:

*“At this point, the government has strong commitment to support girls not to undergo CFM and FGM/C. The administration office is really helping…it has established steering committee from women, youth and child office, health, education, and police to review and make decisions if any cases or issues are identified. So at least, I can say the government is putting a lot of effort to see FGM/C and CFM are not totally practised in our zones… So, the police officer gets information from the community and checks in person for validation of the information to start the legal procedures.”* Policymaker #2, Ethiopia.

In contrast to Ethiopia, qualitative interviews with policymakers and programme implementers in Senegal revealed a focus by the government to contain the spread of COVID-19 through introduction of curfews with limited strategy on prevention or response to FGM/C and CFM.

### Adequacy of the health system in addressing FGM/C and CFM

*To quantitatively assess* the adequacy of the health system in addressing FGM/C, community members were asked to compare services that were offered by the health system before and during COVID-19 (Table 4). Respondents were also asked to rate the health system’s response. In Kenya, there were perceived differences in services offered before COVID-19 and during the pandemic. For example, before COVID-19, services provided for FGM/C cases included psychological and sexual counselling (52%), rescue services (45%) and reintegration back to the community (23%). During COVID-19, there was perceived increase in psychological and sexual counselling (69%), a reduction in rescue services (18%) and reintegration back to the community services (7%), and a remarkable increase in no services offered from 15% (before COVID-19) to 49% (during COVID-19). Generally, over 70% of respondents rated the response of the health system in addressing FGM/C and CFM during the pandemic as either poor or average.

**Table 4:** Health systems and provider’s response to FGM/C cases before and during COVID-19

	Kenya			Uganda			Ethiopia			Senegal		
	**N**	**%**	***P*-value**	**N**	**%**	***P*-value**	**N**	**%**	***P*-value**	**N**	**%**	***P*-value**
**Services provided for FGM/C cases before COVID-19**:												
Psychological and sexual counselling	163	52.2	< 0.001	247	88.8	0.282	238	94	0.74	107	51.4	0.235
De-infibulation	7	2.2	0.011	1	0.4	0.631	0	0.0		7	3.4	0.407
Clitoral reconstruction	1	0.3	0.340	1	0.4	0.631	0	0.0		4	1.9	0.348
No services	48	15.4	< 0.001	15	5.4	0.004	43	17.1	0.798	0	0.0	
Rescue	139	44.6	< 0.001	89	32.0	0.587	0	0.0		7	3.4	0.617
Reintegration back to the community	71	22.8	< 0.001	2	0.7	0.003	50	19.9	0.923	13	6.3	0.73
Don’t know/No response	14	4.5	0.026	1	0.4	0.631	2	0.8	0.484	12	5.8	0.631
Other	0	0.0		2	0.7	0.255	0	0.0		1	0.5	0.521
**Services provided for FGM/C cases during Covid-19**:												
Psychological and sexual counselling	115	69.3	< 0.001	232	93.5	0.032	103	41.0	0.976	117	56.3	0.713
De-infibulation	24	14.5	< 0.001	1	0.4	0.642	21	8.4	0.973	11	5.3	0.196
Clitoral reconstruction	21	12.7	< 0.001	1	0.4	0.642	21	8.4	0.973	10	4.8	0.403
No services	82	49.4	0.002	0	0.0		0	0.0		47	22.6	0.806
Rescue	29	17.5	< 0.001	44	17.7	0.068	22	8.8	0.886	41	19.7	0.038
Reintegration back to the community	11	6.6	0.001	4	1.6	0.003	62	24.7	0.995	57	27.4	0.053
Don’t know/No response	7	4.2	0.031	2	0.8	0.510	114	45.4	0.991	29	13.9	0.731
Other	0	0.0		0	0.0		0	0.0		0	0.0	
**Percentage of respondents rating provider’s response to FGM/C during COVID-19 as**:												
Poor	101	32.4	0.001	46	16.5	0.446	14	5.6	0.772	50	27.5	0.001
Average	136	43.6		56	20.1		25	10		40	22	
Good	59	18.9		172	61.9		149	59.4		50	27.5	
Excellent	6	1.9		4	1.4		59	23.5		26	14.3	
Don’t know/No response	10	3.2		0	0.0		4	1.6		16	8.8	
**Percentage of respondents rating provider’s response to CEFM during COVID-19 as**:												
Poor	106	34.0	< 0.001	36	12.9	0.503	53	32.3	< 0.001	58	31.9	< 0.001
Average	135	43.3		63	22.7		27	16.5		37	20.3	
Good	57	18.3		176	63.3		56	34.1		48	26.4	
Excellent	6	1.9		3	1.1		22	13.4		27	14.8	
Don’t know/No response	8	2.6		0	0.0		6	3.7		12	6.6	
**Total**	**312**	**100.0**		**278**	**100.0**		**251**	**100.0**		**208**	**100.0**	

In Uganda, there were perceived minimal changes in services offered before and during COVID-19. Psychological and sexual counselling which was the most common service offered to FGM/C survivors marginally increased from 89% before COVID-19 to 94% during the pandemic. There was also a decline in rescue services from 32% before COVID-19 to 18% during the pandemic. Slightly over 60% of respondents rated the response of the health system in addressing FGM/C and CFM during the pandemic as good.

In Ethiopia, respondents’ perception of the health system’s response to FGM/C and CFM during the pandemic showed that there was a difference in services offered before COVID-19 and during the pandemic. Specifically, before COVID-19, the most common service provided for FGM/C cases was psychological and sexual counselling (95%) which reduced during the pandemic (41%). There was a slight increase in reintegration of girls back to the community and respondents who reported lack of services during the pandemic. Over 80% of respondents rated the response of the health system in addressing FGM/C during the pandemic as either good or excellent while nearly half of the respondents were of the view that the health system’s response on CFM was either poor or average.

In Senegal, there were perceived minimal differences in services offered before and during the pandemic. Before COVID-19, the most common service provided for FGM/C cases included psychological and sexual counselling (51%) which slightly increased to 56% during the pandemic. Notably, there was an increase in the number of services offered from 0% (before COVID-19) to 23% (during COVID-19). Generally, 48% of respondents rated the response of the health system in addressing FGM/C during the pandemic as either poor or average, while 52% rated its response on CFM as either poor or average.

*Qualitative interviews* with key informants in Kenya showed that the health system had faced challenges in addressing FGM/C and CFM during the pandemic. This was due to frustrations experienced in offering services to the community because of COVID-19 restrictions:

*“This Corona has stopped us so much. We were on the run, planning big things and running around and just having great plans, but all that stopped, and so people are sitting back and just reflecting on their achievements…and just holding unto those achievements…that is a lesson… And then of course… investing in the mental health of human beings is important. That is a lesson that I have learnt.”* Programme Implementer #4, Kenya.

Similar to Kenya, key informants in Uganda underscored the challenges experienced by the health system due to COVID-19. They highlighted the failure by the health system to implement preventive measures at community level due to the limitations posed by COVID-19 restrictions.

In Ethiopia, key informants were of the view that the government had made efforts to ensure that there was coordination between government agencies including the health system. Specifically, the health system had been given the mandate to coordinate multi-agency efforts and make decisions on addressing FGM/C and CFM during COVID-19.

*“The health offices and the police are working closely with us. The health system has established structures starting at grass root level using health extension workers and health development armies to respond to FGM/C and CFM.”* Policymaker #4, Ethiopia.

In Senegal, interviews with programme implementers and policymakers revealed a focus by the ministry of health on containment of the spread of COVID-19 through sanitation and observing social distancing but no strategy on prevention or response to FGM/C and CFM.

### Adequacy of the civil society in addressing FGM/C and CFM

*Quantitative findings* on the adequacy of the civil society’s response in addressing cases of FGM/C and CFM during COVID-19 are shown in Table 5. In Kenya, over 60% of respondents were of the opinion that the civil society’s response to FGM/C and CFM was either poor or average. Some of the alternative approaches used by the civil society to reach victims of FGM/C and CFM during the pandemic included dialogue forums (45%), radio talk shows (40%) and using local champions as part of risk communication (33%).

**Table 5:** Adequacy of the civil society’s response in addressing cases of FGM/C and CEFM during COVID-19

	Kenya			Uganda			Ethiopia			Senegal		
	**N**	**%**	***P*-value**	**N**	**%**	***P*-value**	**N**	**%**	***P*-value**	**N**	**%**	***P*-value**
**Percentage of respondents rating the response of programme implementers to FGM/C cases during COVID-19 as**:												
Poor	69	22.1	< 0.001	62	22.3	0.048	18	7.2	0.651	67	32.2	0.075
Average	135	43.3		42	15.1		30	12.0		46	22.1	
Good	65	20.8		0	0.0		126	50.2		47	22.6	
Excellent	43	13.8		174	62.6		73	29.1		26	12.5	
Don’t know/No response	0	0.0		0	0.0		4	1.6		22	10.6	
**Percentage of respondents rating the response of programme implementers to CEFM cases during COVID-19 as**:												
Poor	68	21.8	0.278	57	20.5	0.032	19	7.6	0.792	37	17.8	0.149
Average	174	55.8		50	18.0		34	13.5		70	33.7	
Good	0	0.0		170	61.2		60	23.9		61	29.3	
Excellent	70	22.4		1	0.4		134	53.4		22	10.6	
Don’t know/No response	0	0.0		0	0.0		4	1.6		18	8.7	
**Total**	**312**	**100.0**		**278**	**100.0**		**251**	**100.0**		**208**	**100.0**	

In Uganda, over 60% of respondents felt that the civil society’s response to FGM/C and CFM was either good (61% on CFM) or excellent (63% on FGM/C). Common alternative approaches used by the civil society to reach victims of FGM/C and CFM during the pandemic was the use of radio talk shows and call centres.

In Ethiopia, over 70% of respondents perceived the civil society’s response to FGM/C and CFM as either good or excellent. Some of the common alternative approaches used by the civil society to reach victims of FGM/C and CFM during the pandemic included use of call centres (62%) and local champions as part of risk communication (33%).

In Senegal, slightly over half of respondents were of the view that the civil society’s response to FGM/C (54%) and CFM (51%) was either poor or average. Alternative approaches used by the civil society to reach victims of FGM/C and CFM during the pandemic included call centres (61%), radio talk shows (51%) and use of local champions as part of risk communication (42%).

*Qualitative interviews* with key informants on the adequacy of the civil society in addressing FGM/C and CFM revealed some of the challenges experienced by organisations working at the community level during the pandemic. In Kenya, key informants highlighted the challenge brought about by the closure of schools which had not only acted as a platform for implementation of interventions but also as a safe space for girls at risk of FGM/C and CFM.

*“If they were in school, we would be able to control because you would have a way of knowing who is here or who has not reported back to school. If they are in school, it is easy to make a report. You see when they are involved with the parent and it is the parents who are encouraging this [FGM/C, CFM] we may not be able to know exactly where the child is.”* Policymaker #6, Kenya.

In Uganda, organisations faced challenges such as restrictions on gatherings which meant field staff could not fully interact with community members to implement behaviour change communication interventions. Reduction in funding towards FGM/C and CFM was also mentioned as a challenge for civil society in addressing FGM/C and CFM during the pandemic.

*“Their funding is not always constant. These guys only come to the field when they receive funding… When they do not have the funding, they don’t implement the activities… I think that is one of the challenges these organizations are facing during COVID-19 as far as implementing activities to end the FGM/C programme is concerned.”* Programme Implementer #6, Uganda.

The situation in Ethiopia was somewhat different whereby key informants were of the opinion that there was good collaboration between the civil society and government agencies in intervening against FGM/C and CFM during COVID-19. Existence of already established structures in monitoring and reporting played a critical role in ensuring synergy between efforts by the government and civil society as exemplified in the following quote:

*“NGOs are working together with health extension workers and health development army structures, the police and judiciary. These are crucial channels for us [NGO] to raise awareness in the community and pass information to the judiciary, health system and the police for further investigation. Non-profit organizations are using these structures to work more efficiently.”* Programme Implementer #6, Ethiopia.

The situation in Senegal was similar to that in Kenya and Uganda. Key informants observed that COVID-19 guidelines on infection prevention disrupted intervention activities directed towards FGM/C and CFM. The directive from government that people needed to stay at home and the introduction of curfews limited implementation of programme activities, interaction with community members and monitoring of FGM/C and CFM.

*“COVID-19 prevention measures were a real obstacle to carrying out awareness-raising activities because one of the instructions was “stay at home” and as a result home visits were no longer being made and therefore people who were no longer being controlled and they did what they wanted to do. What we did was to create awareness activities using community radios.”* Programme Implementer #2, Senegal.

## Discussion

The study aimed to generate evidence on respondents’ perceptions on how COVID-19 pandemic had affected FGM/C and CFM in Kenya, Uganda, Ethiopia, and Senegal. Findings showed that in Kenya, the COVID-19 pandemic contributed to perceived increase in both FGM/C and CFM cases. Closure of schools for longer periods, economic losses and people staying at home for longer periods including potential victims likely contributed to the increase in FGM/C and CFM during COVID-19. In Uganda, the COVID-19 pandemic contributed to perceived minimal increase in FGM/C cases and a significant increase in CFM cases. Economic losses and poverty due to COVID-19 was the most common reason given for perceived increase in CFM cases during the pandemic. The situation in Ethiopia and Senegal showed that the COVID-19 pandemic had limited perceived effect on changes in FGM/C and CFM cases with most of the study respondents believing that the number of FGM/C and CFM cases have remained the same before and during the pandemic. Respondents nonetheless noted that due to the secrecy and stigma associated with these harmful practices, there are challenges of reporting which may mask possible changes.

These findings highlight the importance of schools and educational programmes that serve as safe havens protecting girls against harmful traditional practices such as FGM/C and CFM [23]. They also show how the pandemic has likely exacerbated the practices due to the economic vulnerability which pushes households to engage in any form of income generating activity including marrying off their young daughters to make ends meet. At the beginning of the COVID-19 pandemic, it was observed that while the depth and severity of the pandemic was still uncertain, it was clear that many households in the COVID-19 affected areas experienced some economic shock due to increased unemployment and the shrinking sources of income [16, 24]. The perceived effect of these economic shocks caused by the pandemic may have introduced economic insecurity and increased stress levels to community members due to the uncertainties which propagate negative wellbeing. Evidence shows that in such contexts, economic insecurity can lead to increases in incidence of gender-based violence and violence against children [25–27].

The effect of quarantines that have become common amid the COVID-19 pandemic may also precipitate social effects as evidenced on health emergencies such as SARS, Swine Flu, and influenza. These examples of health emergencies were associated with problematic coping behaviours and psychosocial complications [28–32]. Restriction of movement can be challenging for parenting with increases in abuse meted against children due to a confluence of school closures, stress, fear, and uncertainty [33]. Evidence also shows that quarantines have been generally associated with violence against women and children through increasing their day-to-day exposure to potential perpetrators [27, 34–37].

The impact of COVID-19 on the justice and legal system, health system, and the response of civil society in addressing FGM/C and CFM varied across the four countries illustrating contextual influences. Overall, the pandemic negatively affected implementation of interventions by the justice and legal system, the health system, and civil societies in preventing FGM/C and CFM and offering services to those affected by these harmful traditional practices. Literature on the impact of COVID-19 shows that implementation of interventions by organisations especially at the community level have been significantly hampered by the restrictions put in place to contain the pandemic [38]. Closure of schools led to interruption of programmes that target students while limited funding meant that interventions were suspended. Nonetheless, some organisations have tried to innovate with new approaches to address concerns during the pandemic such as the use of mass media to reach the target population [39].

Evidence from prior epidemics such as Ebola outbreak in Liberia and Sierra Leone show that emergency measures taken to tame the epidemic negatively affected the operation of organisations implementing interventions [40]. Due to changes in priorities with a focus on saving lives from the epidemic, other important sectors of the society such as gender-based violence were neglected accentuating the already fragile context [18, 40]. Evidence has also shown a reduction in the health services availability and access to first responders for women and girls experiencing violence in times of a crisis such as COVID-19 [27]. For example, to contain the SARS outbreak in Toronto, restrictions were put in place on non-urgent hospital services which led to a substantial reduction in elective procedures and non-SARS emergency visits [41]. The contraction of routine health services also affected the screening and service provision for violence against women cases and a reduction in the supply of essential services for victims of violence. It is important to note that in times of a crisis such as a pandemic, the referral pathways may change leading to failure of complementary health and legal services to address existing and emerging needs of women [42, 43].

### Programmatic and research implications

Pandemics are associated with fear and uncertainty, and provide an environment that can exacerbate various forms of violence against women, girls, and children. Given the study findings, the following are the programmatic and research implications:

- Integrate economic empowerment initiatives and social safety net programmes targeting the vulnerable and poor in the society during and after the pandemic as part of journey towards recovery and resilience. Specifically, programmes should target communities that are likely to resort to harmful traditional practices such as CFM for economic benefits. This may include exploring cash transfers, food voucher payments, and tax reliefs that can build resilience and cushion families from the impact of COVID-19.
- Support informal, online, and mobile platforms in reaching women and girls at risk of FGM/C and CFM. There is need to ensure that the online and mobile platforms are safe and accessible as not all women and girls have the privilege to access these platforms. The use of informal support such as friends or family with whom women and girls at risk or survivors may still be in contact with and who may be able to seek help on their behalf is crucial.
- Put in place measures to ensure that legal and judicial officers, healthcare workers and programme implementers have access to all populations in need. There is need to ensure that COVID-19-related movement restrictions are sensitive to the different needs of vulnerable groups in the population such as women and girls at risk of FGM/C and CFM.
- Enhance outreach efforts by using local champions, influential community or religious leaders and community healthcare workers. The focus should be on reinforcing behaviour change communication messaging on the consequences of harmful traditional practices, especially during the pandemic. The approach should ensure that the leadership role and agency of women and girls are visible, and their participation is fully embraced at all levels of community engagement.
- Strengthen relevant government ministries and agencies to enforce instruments and protocols for preventing and ending FGM/C and CFM. This should include putting in place mechanisms to ensure that FGM/C and CFM incidences can be easily monitored and reported during COVID-19. It may also include increasing staff and utilising existing violence prevention and response hotlines and outreach centres.
- Conduct research on gendered implications of public health emergencies such as COVID-19 and effectiveness of alternative approaches used during COVID-19. This will enable evidence generation on public health preparedness and response plans that can mitigate harm to women, girls, and other vulnerable groups. There is need to conduct research on the effectiveness of alternative approaches such as call centres, radio talk shows and the use of local champions as part of risk communication in preventing and responding to FGM/C and CFM during COVID-19.

### Study limitations

Findings from this study should be interpreted with caution bearing in mind the following: First, the study was limited to only four countries in sub-Saharan Africa and therefore findings from these countries cannot be used to generalize for the whole continent. Nonetheless, the four countries represent various regions in sub-Saharan Africa that could highlight unique experiences of COVID-19 on FGM/C and CFM. Second, this study adopted a cross-sectional design in its data collection with a focus on perceptions of respondents on how COVID-19 had affected FGM/C and CFM; interpretation of study findings are therefore limited to that scope. Importantly, due to probable selection bias, study findings cannot be generalized within the countries. However, the study used a mixed methods approach to triangulate our data sources.

## Conclusions

The COVID-19 pandemic had varied perceived effect on FGM/C and CFM across the four focus countries. The pandemic negatively affected implementation of interventions by the justice and legal system, the health system, and civil societies in preventing and responding to FGM/C and CFM. There is need for innovative approaches in intervening in the various communities to ensure that women and girls at risk or in need of services are reached during the pandemic. Alternative approaches such as the use of call centres, radio talk shows and the use of local champions as part of risk communication are already being implemented. Evidence on how effective these alternative approaches are in preventing and responding to FGM/C and CFM amid COVID-19 is required.

## Supplementary Information

**Additional file 1.**

## Data Availability

The datasets used and/or analysed during the current study are available from the corresponding author on reasonable request.

## Abbreviations

FGM/C: Female Genital Mutilation/Cutting
CFM: Child and Forced Marriage
KII: Key Informant Interview
