# Supplementary material for: The perceived effects of COVID-19 pandemic on female genital mutilation/cutting and child or forced marriages in Kenya, Uganda, Ethiopia and Senegal
Source: BMC Public Health. 2022 Mar 29;22:601. doi: 10.1186/s12889-022-13043-w (PMC8961092; doi:10.1186/s12889-022-13043-w)
Supplement: Supplementary file 1 — Additional file 1. [file 12889_2022_13043_MOESM1_ESM.docx]

**Additional file 1**

**Appendix 1: Community survey questionnaire**

| **NO.** | **QUESTIONS AND FILTERS** | | **CODING CATEGORIES** | **SKIP** |  |
| --- | --- | --- | --- | --- | --- |
|  | Do you agree to participate? | | 1=yes | **If No -END INTERVIEW** |  |
|  |  |  | 0=No |  |  |
|  | **Background characteristics** | |  |  |  |
| **100** | Name of country you live in | |  |  |  |
|  |  | |  |  |  |
| **101** | How old are you? (enter in Years*)* | |  |  |  |
| **102** | Respondent lives in | | Urban area | 1 |  |
|  |  |  | Rural settings | 2 |  |
|  |  |  | Peri urban | 3 |  |
| **103** | Record sex of respondent | | Male | 1 |  |
|  |  |  | Female | 2 |  |
|  | **Effect of COVID-19 on number of cases of FGM/C and CEFM** | | | | |
| **104** | For the following questions, please tell me whether the numbers of FGM/C cases are the same or have increased during the pandemic | |  |  |  |
|  | Number of FGM/C cases before COVID-19  were: | Same as now | 1 |  |  |
|  |  | Decreasing | 2 |  |  |
|  |  | Increasing | 3 |  |  |
|  |  | Don’t know, no response | 88 |  |  |
| **105** | Number of FGM/C case during COVID-19 are | Same as before COVID-19 | 1 |  |  |
|  |  | Are decreasing | 2 |  |  |
|  |  | Are increasing | 3 | **If yes, Go to 106** |  |
|  |  | Don’t know, no response | 88 |  |  |
| **106** | What is the main reason would you associate to the increasing number of FGM/C cases? | People are staying at home more including potential victims | 1 |  |  |
|  |  | Reduced efforts in programs supporting potential victims | 2 |  |  |
|  |  | Inability to access support programs due to restrictions of movement | 3 |  |  |
|  |  | Reporting opportunities have become limited | 4 |  |  |
|  |  | Others |  |  |  |
|  |  | Don't know/no response | 88 |  |  |
| **107** | For the following questions, please tell me whether the numbers of CEFM cases are the same or have increased during the pandemic | |  |  |  |
|  | Number of CEFM cases before COVID were: | Same as now | 1 |  |  |
|  |  | Decreasing | 2 |  |  |
|  |  | Increasing | 3 |  |  |
|  |  | Don’t know, no response | 88 |  |  |
| **108** | Number of CEFM cases during COVID are: | Same as before COVID-19 | 1 |  |  |
|  |  | Decreasing | 2 |  |  |
|  |  | Increasing | 3 | **If yes, Go to 109** |  |
|  |  | Don’t know, no response | 88 |  |  |
| **109** | What is the main reason would you associate to the increasing number of CEFM cases? | People are staying at home more including potential victims | 1 |  |  |
|  |  | Reduced efforts in programs supporting potential victims | 2 |  |  |
|  |  | Inability to access support programs due to restrictions of movement | 3 |  |  |
|  |  | Reporting opportunities have become limited | 4 |  |  |
|  |  | Others |  |  |  |
|  |  | Don't know/no response | 88 |  |  |
|  | **Adequacy of the legal system’s response in addressing cases of FGM/C and CEFM during the COVID-19 crisis** | | | | |
| **110** | How would you rate the response of the legal system on FGM/C during the pandemic | Poor | 1 |  |  |
|  |  | Average | 2 |  |  |
|  |  | Good | 3 |  |  |
|  |  | Excellent | 4 |  |  |
|  |  | Don’t know/No response | 88 |  |  |
| **111** | How would you rate the response of the legal system on CEFM during the pandemic | Poor | 1 |  |  |
|  |  | Average | 2 |  |  |
|  |  | Good | 3 |  |  |
|  |  | Excellent | 4 |  |  |
|  |  | Don’t know/No response | 88 |  |  |
| **112** | What has been the main barriers to the justice and legal system to respond effectively during this pandemic | Inadequate reporting by victims | 1 |  |  |
|  |  | Fear of lack of services being offered | 2 |  |  |
|  |  | Challenges of accessing victims due to restrictions | 3 |  |  |
|  |  | It has always not responded | 4 |  |  |
|  |  | Others |  |  |  |
|  |  | Don’t Know/No response | 88 |  |  |
|  | **Adequacy of the health system’s response in addressing cases of FGM/C and CEFM during the COVID-19 crisis** | | | | |
| **113** | What services were being provided for FGM/C cases before COVID-19 in your country | Psychological and sexual counselling | 1 |  |  |
|  |  | De-infibulation | 2 |  |  |
|  |  | Clitoral reconstruction | 3 |  |  |
|  |  | No services | 4 |  |  |
|  |  | Others |  |  |  |
|  |  | Don’t Know/ No response | 88 |  |  |
| **114** | **[If services were being offered before COVID-19]**  As a result of COVID-19 pandemic what services are not being offered for FGM/C cases | Psychological and sexual counselling | 1 |  |  |
|  |  | De-infibulation | 2 |  |  |
|  |  | Clitoral reconstruction | 3 |  |  |
|  |  | Others |  |  |  |
|  |  | Don’t Know/ No response | 88 |  |  |
| **115** | How would you rate the response of the health care workers on FGM/C during the pandemic | Poor | 1 |  |  |
|  |  | Average | 2 |  |  |
|  |  | Good | 3 |  |  |
|  |  | Excellent | 4 |  |  |
|  |  | Don’t know/No response | 88 |  |  |
| **116** | How would you rate the response of the health care workers on CEFM during the pandemic | Poor | 1 |  |  |
|  |  | Average | 2 |  |  |
|  |  | Good | 3 |  |  |
|  |  | Excellent | 4 |  |  |
|  |  | Don’t know/No response | 88 |  |  |
|  | **Adequacy of the civil society’s (CSO, NGO, FBO) response in addressing cases of FGM/C and CEFM during the COVID-19 crisis** | | | | |
| **117** | How would you rate the response of programme implementers on FGM/C cases during COVID-19 | Poor | 1 |  |  |
|  |  | Average | 2 |  |  |
|  |  | Good | 3 |  |  |
|  |  | Excellent | 4 |  |  |
|  |  | Don’t know/No response | 88 |  |  |
| **118** | How would you rate the response of programme implementers on CEFM cases during COVID-19 | Poor | 1 |  |  |
|  |  | Average | 2 |  |  |
|  |  | Good | 3 |  |  |
|  |  | Excellent | 4 |  |  |
|  |  | Don’t know/No response | 88 |  |  |
|  | **Demographics** |  |  |  |  |
| **119** | What is the highest level of education you have completed? | No schooling | 0 |  |  |
|  |  | Incomplete primary school | 1 |  |  |
|  |  | Completed primary school | 2 |  |  |
|  |  | Incomplete secondary school | 3 |  |  |
|  |  | Completed secondary school | 4 |  |  |
|  |  | Incomplete higher education | 5 |  |  |
|  |  | Completed higher education (Tech, college, university) | 6 |  |  |
|  |  | Don't know, no response | 88 |  |  |
| **120** | What is your current marital status? | Married | 1 |  |  |
|  |  | Living together with partner but not married | 2 |  |  |
|  |  | Separated | 3 |  |  |
|  |  | Divorced | 4 |  |  |
|  |  | Widowed | 5 |  |  |
|  |  | Single | 6 |  |  |
|  |  | Don't know, no response | 88 |  |  |
| **121** | The following questions are about items that your household may have or own. You will not be receiving any benefit based on your answers, so we ask you to be as honest as possible. | **NO** | **YES** |  |  |
|  |  | Reliable electricity | 0 | 1 |  |
|  |  | Reliable piped water: in house | 0 | 1 |  |
|  |  | Reliable water: tube well | 0 | 1 |  |
|  |  | Reliable access to handwashing station | 0 | 1 |  |
|  |  | Reliable access to soap | 0 | 1 |  |
| **122** | Do you own your own mobile phone? | No | 0 |  |  |
|  |  | Yes | 1 |  |  |
|  |  | Don't know, no response | 88 |  |  |
| **123** | Does anyone else in your household own a mobile phone that you use (regularly)? | No |  | 0 |  |
|  |  | Yes |  | 1 |  |
|  |  | Don't know, no response |  | 88 |  |
|  |  | **Check boxes** |  |  |  |
| **124** | Now we would like to ask about you source of livelihood before and after COVID-19          Circle categories that apply before and after |  | **Before** | **During COVID-19** |  |
|  |  | Farming | 1 | **1** |  |
|  |  | Petty trade | 2 | 2 |  |
|  |  | Private business | 3 | 3 |  |
|  |  | Unemployed | 4 | 4 |  |
|  |  | Formal employment | 5 | 5 |  |
|  |  | Don't know, no response | 88 |  |  |
|  | **We have reached the end of the interview. Thank you so much for your time and for answering our questions.** | | | | |

**Appendix 2: Key informant interview guide for programme implementers**

Date of interview: [___|___ /___|___/ 2 | 0 | 2 | 0 ]

Time of interview: Start [___|___:___|___] End [___ |___:___|___]

Country: __________________________________________________

Institution: __________________________________________________

Designation: __________________________________________________

Age: __________________________________________________

Gender: __________________________________________________

Thank you for accepting to participate in this interview. Your responses will be helpful in understanding the COVID-19 pandemic has affected FGM/C and CEFM in Africa. The reason for having this interview is to find out your views and experiences with regard to the impact of COVID-19 on FGM/C and CEFM in your country.

1. **Qualifications and experience working in FGM/C and/or CEFM**

- I will start by asking you about your qualifications and experiences working in FGM/C and/or CEFM.
- What is your professional qualification?
- How long have you worked in FGM/C and/or CEFM?
- How long have you worked at this institution?
- What are some of the efforts/interventions you have been involved in related to FGM/C or CEFM?

1. **Views and experiences on the impact of COVID-19 on FGM/C and CEFM cases**

- Let’s now talk about your views and experiences on the impact of COVID-19 on FGM/C and CEFM.
  - How has COVID-19 pandemic affected FGM/C and CEFM? in what way?
    - Numbers of cases
    - Other effects of COVID-19 on FGM/C and CEFM?
  - What are some of the changes you may have observed in FGM/C and CEFM during the COVID-19 pandemic period?
- What is your view about the number of FGM/C cases before and during the COVID-19 pandemic?
- Probe: What are the reasons for the observed changes
- What is your view about the number of CEFM cases before and during the COVID-19 pandemic?
- Probe: What are the reasons for the observed changes

1. **Views on how civil society organisations (NGOs, CBOs, FBOs) have responded to FGM/C and CEFM amid the COVID-19 pandemic**

- Let’s now talk about your views and experiences on civil society have responded to FGM/C and CEFM amid the COVID-19 pandemic.
  - What are some of the strategies that civil society have put in place to address FGM/C and CEFM during the COVID-19 pandemic?
- Probe: Response by NGOs, CBOs and FBOs
  - What are some of the challenges faced by civil society organisations in addressing FGM/C and CEFM during the COVID-19 pandemic?
  - What are your thoughts or recommendations for civil society organisations on the way forward in implementation of FGM/C and CEFM intervention efforts amid the COVID-19 pandemic?
- Probe: Going forward, what can be done by NGOs, CBOs and FBOs to end FGM/C and CEFM amid the COVID-19 pandemic

1. **Views about government response to FGM/C and CEFM amid COVID-19 pandemic**

- Let’s now talk about your views and experiences on how the government has responded to FGM/C and CEFM amid COVID-19 pandemic.
  - What are some of the strategies that government agencies have put in place to address FGM/C and CEFM during the COVID-19 pandemic?
- Probe: Response by the health system, judiciary and police
  - What are some of the challenges faced by government agencies in addressing FGM/C and CEFM during the COVID-19 pandemic?
  - What are your thoughts or recommendations on the way forward in implementation of FGM/C and CEFM intervention efforts amid the COVID-19 pandemic?
- Probe: Going forward, what can be done by the health system, judiciary, and police

1. **Lessons learnt from other similar pandemics such as Ebola Virus Disease in addressing FGM/C and CEFM amid COVID-19 pandemic**

- Let’s now talk about your views and experiences on how the government and other implementing partners can learn from previous epidemics such as Ebola in responding to FGM/C and CEFM amid the COVID-19 pandemic.
  - What are some of the lessons that government agencies and other implementing partners can borrow and implement from previous epidemics such as Ebola in addressing FGM/C and CEFM during the COVID-19 pandemic?
- Probe: Lessons learnt for the health system, judiciary, police, and civil society (NGOs, CBOs, FBOs)

**We have now come to the end of our discussion.**

- Do you feel that there is anything we have left out or is there something you would like to mention regarding the impact of COVID-19 on FGM/C and CEFM in particular or generally?
- Is there any other general issue you would like to raise?

**THANK YOU VERY MUCH FOR YOUR TIME.**

**Appendix 3: Key informant interview guide for policymakers**

Date of interview: [___|___ /___|___/ 2 | 0 | 2 | 0 ]

Time of interview: Start [___|___:___|___] End [___ |___:___|___]

Country: __________________________________________________

Institution: __________________________________________________

Designation: __________________________________________________

Age: __________________________________________________

Gender: __________________________________________________

Thank you for accepting to participate in this interview. Your responses will be helpful in understanding the COVID-19 pandemic has affected FGM/C and CEFM in Africa. The reason for having this interview is to find out your views and experiences with regard to the impact of COVID-19 on FGM/C and CEFM in your country.

1. **Qualifications and experience working in FGM/C and/or CEFM**

- I will start by asking you about your qualifications and experiences working in FGM/C and/or CEFM.
- What is your professional qualification?
- How long have you worked in FGM/C and/or CEFM?
- How long have you worked at this institution?
- What are some of the efforts/interventions you have been involved in related to FGM/C or CEFM?

1. **Views and experiences on the impact of COVID-19 on FGM/C and CEFM cases**

- Let’s now talk about your views and experiences on the impact of COVID-19 on FGM/C and CEFM.
  - How has COVID-19 pandemic affected FGM/C and CEFM? in what way?
    - Numbers of cases
    - Other effects of COVID-19 on FGM/C and CEFM?
  - What are some of the changes you may have observed in FGM/C and CEFM during the COVID-19 pandemic period?
- What is your view about the number of FGM/C cases before and during the COVID-19 pandemic?
- Probe: What are the reasons for the observed changes
- What is your view about the number of CEFM cases before and during the COVID-19 pandemic?
- Probe: What are the reasons for the observed changes

1. **Views about government response to** **FGM/C and CEFM amid COVID-19 pandemic**

- Let’s now talk about your views and experiences on how the government has responded to FGM/C and CEFM amid COVID-19 pandemic.
  - What are some of the strategies that government agencies have put in place to address FGM/C and CEFM during the COVID-19 pandemic?
- Probe: Response by the health system, judiciary, and police
  - What are some of the challenges faced by government agencies in addressing FGM/C and CEFM during the COVID-19 pandemic?
  - What are your thoughts or recommendations on the way forward in implementation of FGM/C and CEFM intervention efforts amid the COVID-19 pandemic?
- Probe: Going forward, what can be done by the health system, judiciary, police, and civil society (NGOs, CBOs, FBOs) to end FGM/C and CEFM amid the COVID-19 pandemic

1. **Lessons learnt from other similar pandemics such as Ebola Virus Disease in addressing FGM/C and CEFM amid COVID-19 pandemic**

- Let’s now talk about your views and experiences on how the government and other implementing partners can learn from previous epidemics such as Ebola in responding to FGM/C and CEFM amid the COVID-19 pandemic.
  - What are some of the lessons that government agencies and other implementing partners can borrow and implement from previous epidemics such as Ebola in addressing FGM/C and CEFM during the COVID-19 pandemic?
- Probe: Lessons learnt for the health system, judiciary, police, and civil society (NGOs, CBOs, FBOs)

**We have now come to the end of our discussion.**

- Do you feel that there is anything we have left out or is there something you would like to mention regarding the impact of COVID-19 on FGM/C and CEFM in particular or generally?
- Is there any other general issue you would like to raise?

**THANK YOU VERY MUCH FOR YOUR TIME.**
